# Microtubule choreography: spindle self-organization during cell division

**DOI:** 10.1007/s12551-024-01236-z

**Published:** 2024-09-30

**Authors:** Amruta Sridhara, Yuta Shimamoto

**Affiliations:** 1 Laboratory of Physics and Cell Biology, National Institute of Genetics, Shizuoka, 411-8540 Japan; 2 The Graduate University for Advanced Studies, SOKENDAI, Shizuoka, 411-8540 Japan

**Keywords:** Microtubule, Motor protein, MAPs, Self-organization

## Abstract

During cell division, the network of microtubules undergoes massive rearrangement to self-organize into the spindle, a bipolar structure essential for accurate chromosome segregation. This structure ensures the stable transmission of the genome from the mother cell to two daughter cells, yet the process by which the ordered architecture emerges from a collection of protein “parts” remains a mystery. In this review, we focus on several key spindle proteins, describing how they move, crosslink, and grow microtubules in vitro and contribute to the spindle’s structural organization. We categorize these proteins into groups, such as transporters, bundlers, and nucleators, to highlight their functional roles. We also present an advanced perspective on the spindle’s complex polymer architecture and its temporal assembly order in cellular contexts. This in situ level information should guide the minimal reconstitution of the spindle, helping to elucidate the biophysical principles underlying essential cytoskeletal self-organization.

## Introduction

**Fig. 1:**
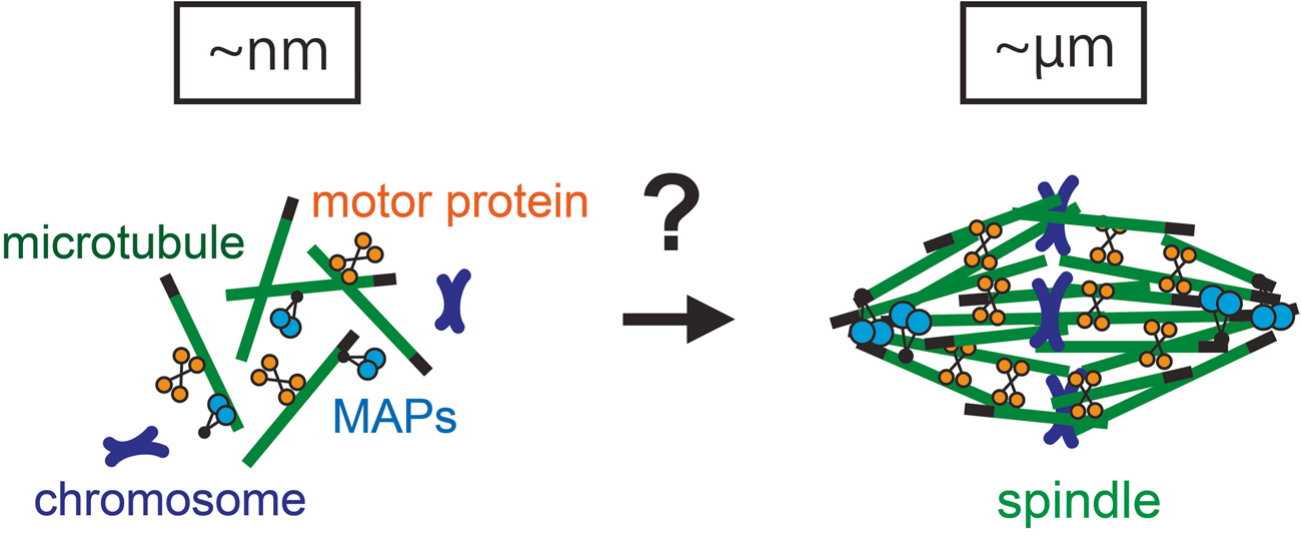
|Spindle self-organization. During cell division, microtubules are self-organized into the spindle, a bipolar machine crucial for chromosome segregation. This complex process involves various microtubule-associated proteins, including motor proteins and non-motor MAPs. The emergence of the ordered, micron-sized architecture from the collection of smaller protein parts remains an area of active research

Microtubules are hollow cylindrical polymers assembled from α- and β-tubulins, forming diverse arrays of architectures in eukaryotic cells (Kirschner and Mitchison 1986; Subramanian and Kapoor 2012; Akhmanova and Steinmetz 2015). A distinct architecture that emerges during cell division is the spindle, which generates nanonewton-order forces to assemble itself and segregate chromosomes into the newly created daughter cells (Fig. 1). The spindle is characterized by its bipolar, football-shaped architecture, directing duplicated chromosomes to the opposite poles of the dividing cell. The spindle also forms at an appropriate size, allowing it to fit within the cell while maintaining enough space for sister chromatid separation. Failures in properly assembling the spindle can lead to chromosome segregation errors, which have been linked to cancer, miscarriage, and congenital disorders (Kops et al. 2005; Gordon et al. 2012; Thomas et al. 2021).

Since the pioneering description of this cytoskeletal structure more than 100 years ago (Flemming 1882), extensive studies have explored its intricate architecture, dynamics, and mechanics (Inoue 1981; McIntosh and Hays 2016; Oriola et al. 2018; Valdez et al. 2023). Our knowledge of the molecules constituting the spindle has also significantly advanced, and we now know many details of their structural, biochemical, and biophysical properties (Compton 2000; Walczak and Heald 2008; Petry 2016; Prosser and Pelletier 2017). Nevertheless, there still exists an unfilled gap between molecular and cellular properties, and we cannot explain how a collection of molecules gives rise to the defined subcellular architecture, whose size is thousands of times larger than their own (Fig. 1). Proteins are nanometer-sized, while spindles are micron-sized. The past decade has seen significant advancements in the development of new tools and approaches, unveiling the mystery of this cytoskeletal choreography.

**Fig. 2:**
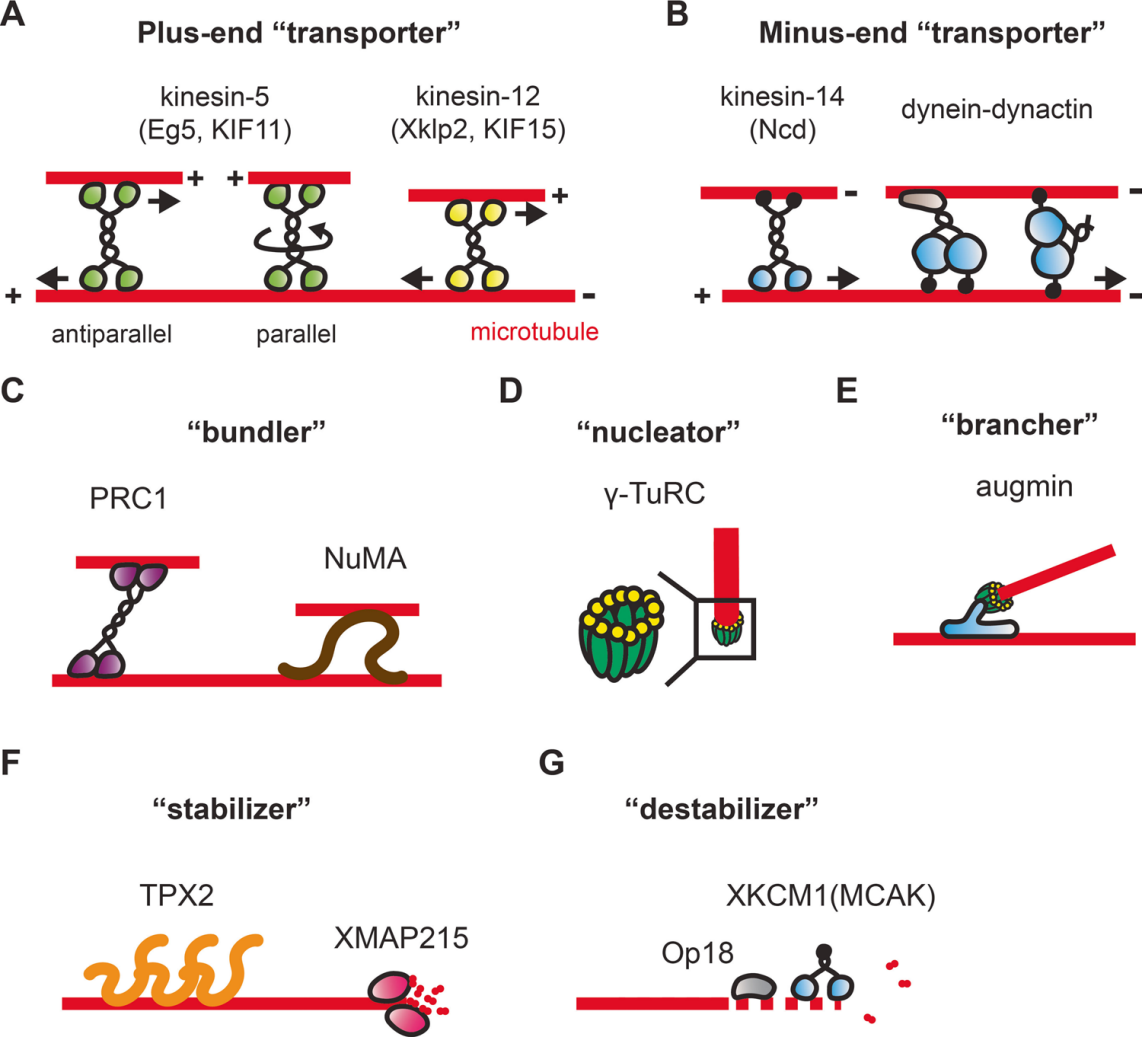
Spindle “parts” list. **A** Microtubule transporters that move toward the plus-end of the polymer. These include kinesin-5 and kinesin-12. Kinesin-5 has a pair of dimeric motor domains at the opposite ends of the dumbbell-shaped molecule, crosslinking antiparallel and parallel microtubules. The semi-circle arrow indicates a torsional twist that is supposed to arise within kinesin-5. Kinesin-12 can be a dimer or a tetramer, crosslinking and sliding microtubules. **B** Microtubule transporters that move toward the minus end of the polymer. These include kinesin-14 and dynein. Kinesin-14 has a diffusive tail that weakly captures the microtubule it carries. Dynein transports microtubules with the dynactin complex. Dynein can also crosslink adjacent polymers on its own using its two motor domains. **C** Microtubule bundlers. These include PRC1 and NuMA, which can crosslink adjacent microtubules and generate frictional drag against active polymer sliding. **D** Microtubule nucleator, γ-TuRC. **E** Microtubule brancher, the augmin complex. **F** Microtubule stabilizers that promote microtubule assembly. These include TPX2 and XMAP215, which bind to either the lattice (shaft) of microtubules or their ends. **G** Microtubule destabilizers that promote microtubule disassembly. These include Op18 and MCAK. Note that some of the proteins have multiple functions. For example, motor proteins can act as frictional brakes against fast polymer sliding; stabilizers can bundle microtubules. Other proteins not listed here also function to build the spindle

Spindle assembly is considered a process of self-organization, in which an ordered structure emerges from integrated local interactions of smaller component parts (Nicolis and Prigogine 1977). Thus far, no template or master organizer that defines spindle size and shape has been identified, except for some potential instructional cues such as centrosomes and chromosomes. This framework motivated researchers to reconstitute the spindle by mixing purified components. Indeed, some protein mixtures successfully reproduced characteristic microtubule architectures, such as parallel and antiparallel arrays and radial asters, which are recurring motifs of the spindle (Surrey et al. 2001; Kapitein et al. 2005; Fink et al. 2009; Hentrich and Surrey 2010; Bieling et al. 2010; Dogterom and Surrey 2013; Roostalu et al. 2018). However, no such components have yet given rise to the steady bipolar spindle. Most of the time, mixtures of components tend to fall apart, form non-discrete polymer arrays, or end up with unipolar architectures. What are the missing parts for the minimal reconstitution of the spindle? In this review, we highlight key spindle “parts” that have been characterized for their molecular and ensemble properties (Fig. 2), aiming to find a way to build the minimal spindle. Achieving spindle reconstitution would allow us to determine the fundamental principles of cytoskeletal self-organization. Our focus here is on metazoan spindles, which are tens of microns in size and comprise thousands of microtubules.

### Transporters

Motor proteins are a class of enzymes that hydrolyze ATP and perform mechanical work. The forces exerted are crucial for constructing the spindle as they push and pull microtubules and transport the polymers to proper subcellular locations. Among many, some of the best-studied motor proteins include kinesin-5, kinesin-14, and dynein, while myosins and other motors also play important roles. Inhibition of motor proteins can lead to aberrant spindle phenotypes, suggesting that they are essential “parts” for spindle assembly.

Kinesin-5 is a homotetrameric motor protein with pairs of microtubule-binding, dimeric ATPase “heads” at the opposite ends of the dumbbell-shaped molecule (Fig. 2A). Inhibition of kinesin-5 results in the spindle collapsing into a monopolar shape (Mayer et al. 1999; Kapoor et al. 2000), demonstrating the crucial role of this motor in building the bipolar spindle. Kinesin-5’s individual dimers walk toward the growing plus-end of the microtubules that it crosslinks, resulting in antiparallel microtubules being pushed apart with their minus-ends leading. This motile property was first demonstrated using an elegant in vitro reconstitution assay (Kapitein et al. 2005), which employed biotinylated microtubules immobilized on a passivated coverslip surface to form overlaps with non-biotinylated microtubules, which were free from the surface. GFP-tagged kinesin-5 molecules were used to crosslink the overlapping microtubules, and a relative sliding was observed in the presence of ATP. Because the velocity of the microtubule sliding nearly doubles the speed of kinesin-5 moving on single microtubules, it was concluded that this homotetrameric motor protein uses both dimers to simultaneously walk toward microtubule plus-ends and push the microtubules apart (Kapitein et al. 2005). The assay further showed that the carboxy-terminal tail of kinesin-5 acts as a diffusive tether on microtubules and plays a role in switching motor activity upon polymer crosslinking (Kapitein et al. 2008; Weinger et al. 2011; Bodrug et al. 2020). Building on this assay, optical tweezers were used to measure forces generated by kinesin-5 pushing apart two microtubules (Shimamoto et al. 2015). The results show that kinesin-5 generates a collective pushing force whose magnitude increases with the extent of antiparallel microtubule overlap. The number of kinesin-5 molecules also increases with the extent of the overlap, suggesting that more kinesin-5 molecules are recruited to longer overlaps, generating an additive pushing force. The additive nature of kinesin-5 force is in contrast to that of kinesin-1 carrying cargo along a microtubule track, in that the force is nearly constant regardless of the total number of motors (Furuta et al. 2013). The additive nature also explained how the spindle controls its size in a cell (Shimamoto et al. 2015). Importantly, analysis of parallel microtubule pairs showed that the force exerted by kinesin-5 is significantly suppressed regardless of the extent of polymer overlap while the motors engage in a tight crosslink between the two microtubules. The mechanism of this suppression is not yet clear, but one possibility is that the motors become inactivated by a tortional twist that arises within the dumbbell-shaped molecule, as both heads within the homotetramer achieve stereospecific interaction with the microtubules (Fig. 2A). In flies, kinesin-5 preferentially crosslinks antiparallel over parallel microtubules (Wildenberg et al. 2008), while no such preference has been found in frogs (Hentrich and Surrey 2010; Shimamoto et al. 2015). Remaining open questions include the following: (i) how and whether the two dimers at the opposite ends of kinesin-5, which are separated by ~ 80 nm (Kashina et al. 1996; Acar et al. 2013), work in allostery to push apart microtubules, and (ii) whether the activity of kinesin-5 depends on torsional twist arising within the molecule while crosslinking angled microtubule pairs. Anyhow, these past studies demonstrate that the activity of kinesin-5 can be regulated depending on the geometry of the microtubule network, providing an additional layer of regulation beyond biochemical control such as one accompanying phosphokinases (Waitzman and Rice 2014).

Kinesin-14 is another class of kinesin motor protein that forms a homodimer and has a dimeric motor domain at one end of the molecule (Fig. 2B). A distinctive feature of this kinesin is its movement towards the minus-end of microtubules, clustering the polymer ends to form radial asters that resemble a spindle pole (Hentrich and Surrey 2010). Kinesin-14 has a tail domain located distal to the dimeric motor domain, which captures the second microtubule with a weak, diffusive affinity for transport (Reinemann et al. 2018). Although the processivity of kinesin-14 seems to be low compared to other kinesins (Fink et al. 2009; Furuta et al. 2013), a motor ensemble can steadily push apart microtubules with their plus-ends leading (Fink et al. 2009; Reinemann et al. 2018). In addition to its opposite directionality compared to kinesin-5, kinesin-14 drives relative microtubule sliding whose velocity slows down as polymers slide apart and the overlap length decreases (Braun et al. 2017). A recent study also reported that kinesin-14 induces helical sliding of microtubules (Mitra et al. 2020). Since the spindle exhibits a chiral symmetry and likely experiences tortional stress in a cell (Novak et al. 2018), the off-axis movement of kinesin-14 may contribute to this spindle’s mechanical twist. More recently, kinesin-5 was also found to exhibit helical microtubule sliding (Meißner et al. 2024). Whether the twisting of the microtubule network is a prerequisite for proper spindle assembly remains an open question. Kinesin-14 has a nuclear localization signal and localizes within the nucleus before the onset of cell division (She and Yang 2017). Precise control over the temporal dynamics of this motor activity may also be important for proper spindle assembly.

Dynein is another class of minus-end directed motor protein that plays a pivotal role in spindle assembly (Fig. 2B). Dynein functions in conjunction with the dynactin complex, and disruption of this protein complex results in splayed spindle poles (Burkhardt et al. 1997). In cells, dynein’s activity is particularly important for spindle assembly with and without centrosomes (Vaisberg et al. 1993; Merdes et al. 1996; Quintyne et al. 2005). Dynein also pulls the spindle to control chromosome positioning and cell division orientation (Kiyomitsu 2019). Studies have also shown that dynein carries kinesin-5 toward the spindle pole and influences kinesin-5 localization (Uteng et al. 2008). Despite its significance, molecular behaviors of dynein within microtubule networks, particularly in relation to spindles, have been poorly explored in vitro. Pioneering studies have demonstrated dynein-mediated relative microtubule sliding (Tanenbaum et al. 2013; Chakraborty et al. 2020). The assay suggested that sliding involves multiple dynein molecules, whose dimeric motorheads bind to each of the two microtubules they crosslink, rather than using their tails (Tanenbaum et al. 2013) (Fig. 2B). Hence, dynein may employ a mechanism similar to kinesin-5 for microtubule crosslinking and to kinesin-14 for microtubule sliding.

Many cell-based assays suggest that a balance of forces between opposing motor activities is crucial for assembling a stable bipolar spindle (Mitchison et al. 2005; Tanenbaum et al. 2009; Goshima and Scholey 2010; Olmsted et al. 2014; Sturgill et al. 2016; Neahring et al. 2021). However, when opposing motors, such as kinesin-5 and kinesin-14, are mixed in vitro, they often exhibit significant mechanical instability instead of forming a steady microtubule overlap, most likely due to stochastic variations in motor activity (Tao et al. 2006; Hentrich and Surrey 2010). Adding components can suppress microtubule dynamics, but has not yet compensated for this architectural instability (Roostalu et al. 2018). This suggests that the mechanical properties of motor protein ensembles must be finely tuned to achieve a static force balance, or that a more dynamic force balance is required for spindle assembly.

### Bundlers

Non-motor microtubule-associated proteins (MAPs) also play crucial roles in spindle assembly. Some of these MAPs bind to the “tips” of microtubules to regulate polymer growth and stability. Other MAPs decorate the microtubule lattice, affecting polymer stability and/or crosslinking adjacent polymers. Crosslinked microtubules can experience passive frictional drag when motor proteins slide them apart. The balanced mechanical activity between motor and non-motor MAPs is crucial for proper spindle assembly, despite the continuous and seemingly wasteful dissipation of energy fueled by ATP and GTP.

PRC1 is a member of the MAP65 protein family and can crosslink overlapping microtubules (Fig. 2C). Knockdown of PRC1 can lead to failure in assembling antiparallel microtubule arrays, causing spindles to fall apart during anaphase (Mollinari et al. 2002). PRC1 works with motor proteins kinesin-4 and kinesin-5 (Subramanian et al. 2010, 2013). When mixed in vitro with kinesin-4, a plus-end-directed motor protein, PRC1 collaborates with this motor to form a micron-sized “tag” at the growing microtubule tips (Subramanian et al. 2013). This end tag serves as a marker that helps control microtubule stability. The interaction between PRC1 and kinesin-4 also stabilizes antiparallel microtubule overlaps, preventing the spindle from disassembly (Subramanian et al. 2013). In contrast, when mixed with kinesin-5, PRC1 accumulates at antiparallel microtubule overlaps without generating significant mechanical resistance (Subramanian et al. 2010). Although PRC1 has the capacity to generate frictional force (Gaska et al. 2021), microtubule pairs crosslinked by kinesin-5 are not stabilized and continue to slide apart. Therefore, PRC1 has a dual role within microtubule bundles, and its function switches depending on the motor protein partner. The mechanism behind this switching remains to be determined, but it may be influenced by the different heights of motor and non-motor MAPs (i.e., the end-to-end length of the molecules), which affect their affinity within microtubule bundles while competing for polymer spacing (Fig. 2A–C), similar to actin-based muscle sarcomeres (Millman 1998; Shimamoto et al. 2007). In addition to direct intermolecular interactions, the geometry of microtubule bundles, particularly inter-filament spacing, may be key to understanding emergent spindle mechanics.

NuMA is a ~ 230 kDa protein whose localization is predominantly nuclear during interphase and at the spindle pole during cell division (Fig. 2C) (Merdes et al. 1996; Radulescu and Cleveland 2010). NuMA forms large cytoplasmic aggregates with dynein-dynactin and streams poleward along microtubule fibers (Merdes et al. 1996, 2000). NuMA can also localize to spindle poles independently of dynein (Hueschen et al. 2017). NuMA forms liquid-like condensates (Sun et al. 2021) and assembles into an insoluble “matrix” that encapsulates the spindle (Radulescu and Cleveland 2010). In vitro studies have thus far been limited due to the complex behaviors of purified NuMA, though some studies have successfully measured its mechanical properties on microtubules (Forth et al. 2014; Chang et al. 2017). Our current knowledge of NuMA’s biophysical properties largely comes from assays using short fragments. The full-length NuMA may exhibit distinct mechanical behavior, which could be revealed by achieving better biochemical control over the full-length construct in vitro.

### Nucleators

The γ-tubulin ring complex (γ-TuRC) is a large macromolecular complex composed of more than 30 subunits, including γ-tubulin, and serves as the site for microtubule nucleation (Fig. 2D). Initially identified of γ-tubulin through genetic screening in fungi, γ-TuRC was found to have both centrosomal and non-centrosomal functions in microtubule nucleation (Oakley et al. 2015). Ultrastructural analyses using advanced electron microscopy have elucidated the detailed molecular architecture of γ-TuRC (Mammri and Conduit 2024). Specifically, γ-TuRC appears as a corn-shaped configuration with an array of 14 γ-tubulin molecules arranged in a spiral, staircase-like manner at its wide-open surface, providing the site of nucleation for α- and β-tubulins. The side of the complex consists of subunits named GCP2-6, which not only provide an asymmetric scaffold but also interact with other molecular partners to control subcellular localization (Wieczorek et al. 2020). The position of the 14-th γ-tubulin partially overlaps with the 1-st γ-tubulin. γ-TuRC becomes compacted when α- and β-tubulins stack onto the γ-tubulin staircase (Brito et al. 2024), being matched to the canonical 13-protofilament microtubules in mammalian cells. γ-TuRC also “caps” microtubule minus-ends independent of its nucleation activity (Berman et al. 2023). An elegant in vitro assay demonstrated that γ-TuRC is displaced by CAMSAP, another minus-end binder, at the capped microtubule end (Rai et al. 2024). Microtubules that lose γ-TuRC may be stabilized by CAMSAP or may become prone to depolymerization. How microtubules are destabilized in the spindle to compensate for their overall outgrowth remains unknown. Determining which end of the microtubules is depolymerized in the spindle is an important open question and is likely crucial for guiding spindle reconstitution in vitro.

### Branchers

Augmin is a hetero-octameric protein whose knockdown results in aberrant spindle morphology (Goshima et al. 2008) (Fig. 2E). Its Y-shaped molecular configuration allows augmin to bind to the lattice of a pre-existing microtubule and nucleate a new microtubule by recruiting γ-TuRC (Hsia et al. 2014; Song et al. 2018; Zhang et al. 2022). This templated growth leads to the formation of a branched microtubule network and amplifies the total number of microtubules in the spindle (Goshima and Kimura 2010; Petry et al. 2011). Because the angle of microtubule branching by augmin is restricted to less than 30° (Petry et al. 2013), new microtubules form from pre-existing microtubules at shallow angles, allowing the architecture of the microtubule network to be “relayed” from older to newer microtubules. Many microtubules in the spindle turnover on the order of tens of seconds (Salmon et al. 1984; Needleman et al. 2010). Augmin’s branching activity is likely crucial for maintaining the spindle architecture while preserving the dynamic nature of individual polymers. Following branching, augmin is transported toward the microtubule minus-ends, forming radial asters (Scrofani et al. 2024). The robust formation of spindle poles may also rely on the branching activity of augmin.

### Stabilizers

Microtubules in the spindle continuously grow and shrink, and changes in their dynamics impact both the number and length of the polymers. MAPs regulate polymer stability by binding to the growing tips and the shaft of microtubules.

TPX2 is a microtubule-binding protein with a largely disordered structure (Fig. 2F). Both overexpression and depletion of TPX2 lead to abnormally shaped spindles, such as multipolar spindles (Gruss and Vernos 2004; Aguirre-Portolés et al. 2012), suggesting that precise regulation of TPX2 activity is crucial. The cytoplasmic amount of TPX2 also influences spindle size (Helmke and Heald 2014). In vitro studies have revealed that TPX2 has multiple functions in microtubule assembly. For example, TPX2 promotes nucleation and stabilizes microtubules (Schatz et al. 2003; Wieczorek et al. 2015; Reid et al. 2016). TPX2 also forms bundles (Schatz et al. 2003), an activity likely facilitated by its multiple microtubule-binding domains or through oligomerization (Zhang et al. 2017; Guo et al. 2023). Additionally, TPX2 interacts with kinesin-12 and kinesin-5, serving as a regulator for spindle motor proteins (Wittmann et al. 2000; Eckerdt et al. 2008; Gable et al. 2012). Although originally identified as a targeting protein for XKlp2 (Xenopus kinesin-12), the role of TPX2 in motor protein regulation has been little explored in vitro, with only a few studies available (Ma et al. 2011; Drechsler et al. 2014). TPX2 also phase-separates in a crowded environment and accumulates tubulins for polymerization (King and Petry 2020). The interplay between these various propensities of TPX2 in controlling robust spindle assembly remains unclear. An important question is how TPX2 stabilizes microtubules to promote spindle assembly while allowing motor proteins to walk and push–pull the polymers.

XMAP215 is a microtubule polymerase that accelerates microtubule growth (Fig. 2F). As a processive polymerase at the growing plus-end of microtubules, XMAP215 promotes polymer growth by binding tubulin dimers through its TOG (tumor overexpressed gene) domain (Brouhard et al. 2008; Al-Bassam and Chang 2011). XMAP215 also protects growing microtubule ends from destabilization, which can be induced by depolymerizing kinesins such as XKCM1 (Kinoshita et al. 2002) (Fig. 2G). By systematically engineering XMAP215 with varying levels of polymerase activity, researchers have demonstrated a correlation between XMAP215 activity, microtubule growth, and spindle size while maintaining microtubule density and spindle shape (Reber et al. 2013). XMAP215 contains several distinct domains responsible for different aspects of its function and interacts with other microtubule-associated proteins (Al-Bassam and Chang 2011). While its primary function is to accelerate polymer growth, XMAP215 can also cooperate with γ-TuRC to promote microtubule nucleation (Thawani et al. 2018; Liu et al. 2021).

In addition to the myriad of spindle “parts” described above, many other molecules are crucial for spindle assembly, and their perturbation results in assembly failure in cells. These include kinesin-12 (Drechsler et al. 2014), KIFC3 (Hata et al. 2019), Op18 (Cassimeris 2002), CLASP (Al-Bassam and Chang 2011), HURP (Koffa et al. 2006) and katanin (McNally and Vale 1993; Quarmby 2000). Some of these molecules act redundantly to organize the spindle, while others act antagonistically (Tao et al. 2006; Tanenbaum et al. 2009; Hentrich and Surrey 2010; Olmsted et al. 2014; Sturgill et al. 2016). Furthermore, individual spindle “parts” can have multiple functions. For instance, motor proteins can act as a microtubule polymerase or depolymerase (Chen and Hancock 2015; Strothman et al. 2019; Ogren et al. 2022). The essentiality of each component depends on the specific physiological context in which it operates. A bottom-up approach will be instrumental in determining the minimally sufficient conditions for spindle assembly.

## Learning from in vivo

While in vitro approaches provide foundation to explore the principles of spindle self-organization, the reconstituted architectures still fall short of mimicking the true spindle entity. To fully understand the underlying mechanisms, it is crucial to study the processes occurring in a cellular context.

The first thing we would like to discuss is the role of the intracellular chemical gradient of Ran. Ran is a GTPase that controls molecular shuttling between the cytoplasm and the nucleus during interphase, but it creates a spatial gradient around mitotic chromosomes during cell division (Kalab and Heald 2008). The gradient arises from the local activation of Ran to its GTP-bound form by RCC1, a Ran GEF (Guanine nucleotide Exchange Factor) tethered to chromosomes, which facilitates the exchange of GDP for GTP in Ran. Once activated, GTP-bound Ran dissociates from the chromosomes and diffuses into the cytoplasm while it hydrolyzes GTP. During this process, Ran interacts with importin-α and importin-β, leading to the release of key microtubule-stabilizing factors like NuMA and TPX2 near the chromosomes. Ran also directly activates augmin for microtubule branching (Ustinova et al. 2023). Experiments using *Xenopus* egg extracts, the system described in the following paragraph, have shown that Ran-induced microtubule asters can recapitulate certain aspects of spindle assembly (Kalab et al. 2002; Tsai et al. 2003). A biochemical environment that locally stabilizes microtubule dynamics should be essential for achieving a robust spindle assembly while preventing excessive polymer growth across the entire reaction space.

**Fig. 3:**
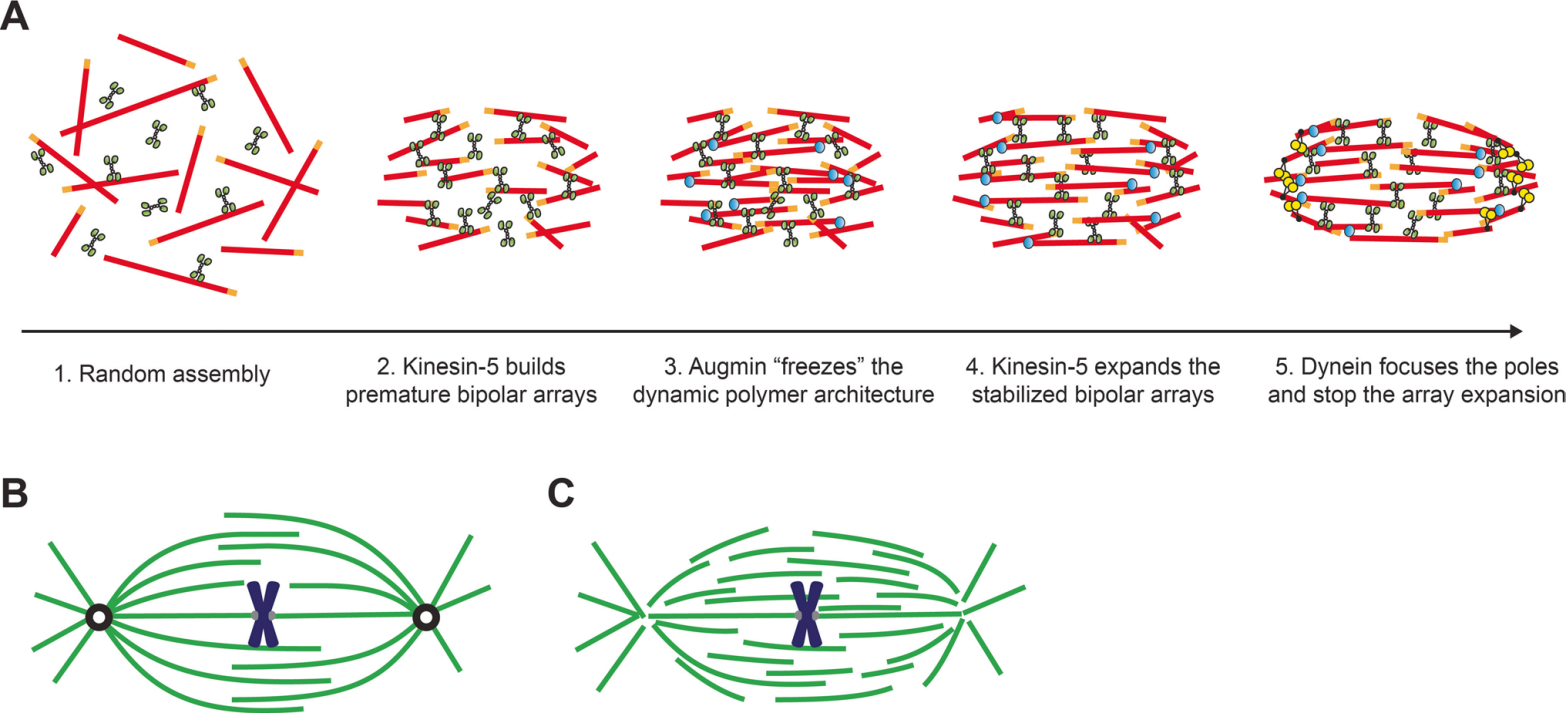
Spindle architecture and self-organization steps. **A** Spindle self-organization steps. The sequence of spindle self-organization is depicted based on high-resolution imaging studies in cytoplasmic extracts (Fukuyama et al. 2022). The figure illustrates three key proteins, kinesin-5 (green), augmin (blue), and dynein (yellow) along with microtubules (red), since experimental perturbations to these components result in a severe spindle defect. Chromosomes are omitted in the drawing. **B**, **C** Spindle architectures. A classic model describes spindles with long microtubules extending radially from the opposite poles and merging at the equator (**B**). An advanced model describes spindles with numerous short microtubules overlapping at various locations. Their minus-ends are dispersed across the spindle structure (**C**)

In a locally stabilized biochemical environment, microtubules begin to assemble the spindle. A powerful experimental platform for quantitatively analyzing the spindle assembly process is the *Xenopus* egg extract system (Desai et al. 1998; Hannak and Heald 2006). This extract, an undiluted “active” cytoplasm prepared from unfertilized eggs, enables spindle assembly in test tubes and offers diverse physical manipulations and high-resolution imaging. Our laboratory has recently utilized this system to visualize and quantify the process of spindle self-organization, i.e., how the ordered structure emerges from microtubule “aggregates.” Through machine learning and quantitative morphology tracking, we have delineated the temporal sequence of spindle self-organization (Fukuyama et al. 2022). Key steps identified include the following: (i) the random assembly of microtubules around condensed chromosomes, (ii) the premature formation of bipolar microtubule arrays by motor proteins, (iii) the stabilization of the bipolar array architecture through polymer branching, (iv) the expansion of the stabilized architecture, and (v) the completion of the structure with pole focusing and counter force balancing (Fig. 3A). The initial bipolarization at steps (ii) and (iii) is particularly important for shaping the spindle into a bipolar structure. Failures in these steps, resulting in broken two-fold symmetry, lead to spindles that exhibit persistent, large shape fluctuations and ultimately develop into faulty multipolar structures (Fukuyama et al. 2022). Such mechanical instability is likely common in eggs and oocytes (So et al. 2022). Although individual microtubules grow and shrink dynamically, correcting an emergent spindle shape is infrequent. In other words, the spindle exhibits multiple steady states, each defining a distinct shape morphology and rarely allowing phenotype switching (Fukuyama et al. 2022). A similar concept was recently suggested through an *in-silico* approach (Li et al. 2023). Our molecular perturbation assays further highlight the roles of augmin and kinesin-5 at each self-organization step (Fig. 3A). We anticipate that such a quantitative approach, combined with molecular perturbations, will enable us to write a comprehensive building instruction of the spindle.

Another important aspect of spindle assembly is its final architecture. Textbooks often depict spindles as having long microtubules extending from two opposite poles and merging at the equator. The spindle architecture is maintained by equatorial microtubule arrays, which are crosslinked by motor and non-motor proteins and slide apart to control spindle size (Fig. 3B). Over the past decade, our understanding of spindle architecture has evolved significantly. Current major models describe spindles, particularly those of metazoans, as composed of a heterogeneous network of microtubules (Yang et al. 2007, 2008; Dumont and Desai 2012; Helmke et al. 2013; Redemann et al. 2017; Müller-Reichert et al. 2018; Takagi et al. 2019; Conway et al. 2022) (Fig. 3C). Specifically, many microtubules in the spindle are short (shorter than the half-spindle length), their minus-ends are not anchored to the poles but are scattered throughout the structure, and individual polymers overlap in parallel and antiparallel arrangements at varying locations within the spindle. This complex architecture enables the spindle to maintain its equatorial microtubule arrays while altering spindle length by sliding parallel microtubules further from the equator, enabling the structure to be adaptable in size and robust in chromosome segregation (Takagi et al. 2019; Valdez et al. 2023). While the classic, simplified depiction of spindle architecture might set an initial milestone for in vitro reconstitution, the more intricate architecture encoding functions should be considered as an ultimate goal.

Before concluding, we would like to discuss insights from polymer physics. Flexible polymers can form a range of ordered patterns, such as nematic and smectic orders; nematic order, for instance, resembles the aligned microtubule architecture in the spindle. Nematic order can emerge when polymers are packed in a confined space (Huber et al. 2013), and also when polymers are exposed to a crowded environment and undergo phase separation (Fu et al. 2024). The phase-separated polymer condensate can form a fusiform-shaped structure called a tactoid (Fu et al. 2024), which mirrors the shape of the mitotic spindle. Given that the cytoplasm is an extremely crowded environment, a tactoid-like condensation of microtubules could be promoted to form or at least to help assemble the spindle. It has been shown that the spindle behaves as an active liquid crystal (Oriola et al. 2018, 2020). A mixture of MAPs and microtubules can generate ordered structures in vitro (Roostalu et al. 2018; Edozie et al. 2019). Considering purely physical forces, such as surface tension and nematic elasticity, is key to ultimately understanding this complex machinery.

## Perspectives

Decades of research have elucidated spindle self-organization at multiple hierarchical levels, from molecular to systemic. Despite significant progress in reconstituting modular spindle architectures in vitro, the fundamental principles of spindle self-organization remain elusive. Over a hundred different types of proteins comprise the spindle and many of them influence the spindle architecture and morphology. This might mean that we need all the proteins in action to reconstitute the spindle. However, there is an optimism that some key proteins act redundantly, and others act antagonistically, allowing for stable spindle assembly through simultaneous inhibition. We anticipate that a combination of top-down and bottom-up approaches will bridge the existing knowledge gaps and provide a definite answer that brings an essential understanding of this remarkable cytoskeletal choreography.

## Data Availability

No datasets were generated or analysed during the current study.
